# Development and Validation of a Job Exposure Matrix for Physical Risk Factors in Low Back Pain

**DOI:** 10.1371/journal.pone.0048680

**Published:** 2012-11-12

**Authors:** Svetlana Solovieva, Irmeli Pehkonen, Johanna Kausto, Helena Miranda, Rahman Shiri, Timo Kauppinen, Markku Heliövaara, Alex Burdorf, Kirsti Husgafvel-Pursiainen, Eira Viikari-Juntura

**Affiliations:** 1 Centre of Expertise for Health and Work Ability, Finnish Institute of Occupational Health, Helsinki, Finland; 2 Disability Prevention Centre, Finnish Institute of Occupational Health, Helsinki, Finland; 3 Centre of Expertise for Human Factors at Work, Finnish Institute of Occupational Health, Helsinki, Finland; 4 Occupational Health Services, OP-Pohjola Group, Helsinki, Finland, and Department of Health Sciences, University of Tampere, Tampere, Finland; 5 Surveillance and Reviews Centre, Finnish Institute of Occupational Health, Helsinki, Finland; 6 National Institute for Health and Welfare, Helsinki, Finland; 7 University of Rotterdam, Rotterdam, The Netherlands; Catholic University of Sacred Heart of Rome, Italy

## Abstract

**Objectives:**

The aim was to construct and validate a gender-specific job exposure matrix (JEM) for physical exposures to be used in epidemiological studies of low back pain (LBP).

**Materials and Methods:**

We utilized two large Finnish population surveys, one to construct the JEM and another to test matrix validity. The exposure axis of the matrix included exposures relevant to LBP (heavy physical work, heavy lifting, awkward trunk posture and whole body vibration) and exposures that increase the biomechanical load on the low back (arm elevation) or those that in combination with other known risk factors could be related to LBP (kneeling or squatting). Job titles with similar work tasks and exposures were grouped. Exposure information was based on face-to-face interviews. Validity of the matrix was explored by comparing the JEM (group-based) binary measures with individual-based measures. The predictive validity of the matrix against LBP was evaluated by comparing the associations of the group-based (JEM) exposures with those of individual-based exposures.

**Results:**

The matrix includes 348 job titles, representing 81% of all Finnish job titles in the early 2000s. The specificity of the constructed matrix was good, especially in women. The validity measured with kappa-statistic ranged from good to poor, being fair for most exposures. In men, all group-based (JEM) exposures were statistically significantly associated with one-month prevalence of LBP. In women, four out of six group-based exposures showed an association with LBP.

**Conclusions:**

The gender-specific JEM for physical exposures showed relatively high specificity without compromising sensitivity. The matrix can therefore be considered as a valid instrument for exposure assessment in large-scale epidemiological studies, when more precise but more labour-intensive methods are not feasible. Although the matrix was based on Finnish data we foresee that it could be applicable, with some modifications, in other countries with a similar level of technology.

## Introduction

Low back pain (LBP) is the most common musculoskeletal complaint causing work-related disability and sickness absence [1], [2]. The annual prevalence of low back pain has ranged between 25% and 60%. Every fourth worker in Europe reports that their work causes back pain [3]. In certain sectors of industry and in some occupations, however, the prevalence of low back pain is considerably higher than in the general working population [4]. Back disorders occur excessively among agricultural, construction, manufacturing, and wholesale workers, as well as among nurses and cleaners [5], [6].

High physical workload, especially manual material handling, frequent bending and twisting of the trunk, and whole-body vibration, have most often been suggested as risk factors for back pain [7], [8]. However, contradicting results on the role of physical workload in back pain have also been reported [9]–[11]. A part of the contradiction is likely due to inaccurate exposure assessment methods. Thus, in order to more reliably estimate the effect of work-related exposures on low back pain, valid and feasible exposure assessment methods are needed. In large epidemiological studies, objective or in-depth assessment of physical work exposure, such as assessment obtained by observation or direct measurement, is often not feasible due to the high costs and time required for data collection. Therefore, exposure information is mainly collected using workers' self-reports (e.g. questionnaires, interviews, and checklists), which are more prone to biases as compared to other methods [12].

The differences between sexes found in work-related musculoskeletal disorders indicate that, in the same occupation, women and men may be exposed differently. It is also possible that they are strained differently while performing the same tasks [13]. Therefore, in order to increase the accuracy of exposure estimates, gender-specific information is necessary.

Job exposure matrices (JEMs) are promising tools for assessing occupational exposures in large epidemiological studies, as well as in studies including occupational titles, but lacking information on individual exposures [14]. Traditionally, such exposure matrices have been developed for chemical and microbiological exposures fairly successfully. Nevertheless, only few of them include estimates of physical workload factors [15], [16].

The objectives of the study were: (1) to construct a gender-specific job exposure matrix for physical risk factors in a representative sample of the Finnish working population, and (2) to evaluate the validity of the matrix in another representative sample of the Finnish working population. We furthermore tested the predictive validity of the matrix against low back pain by comparing the associations of the group-based (matrix) variables with those of individual-based exposures.

## Materials and Methods

### Study population

We utilized two large Finnish population samples. The Health 2000 Study (H2000) was used to construct the JEM and the Finnish National Work and Health Surveys (FWH) to test the validity of the matrix. In the current study the target population consisted of 18–64 year-old individuals, who had been working during the preceding 12 months.

The Health 2000 Study (H2000 Study) was carried out in 2000–2001. The main objective of the study was to obtain up-to-date information on the population's health in Finland. A nationally representative sample of the population was obtained using a two-stage stratified cluster sampling design. The original sample consisted of 8,028 subjects aged 30 years or over. In total, 6986 (87.6%) subjects were interviewed. In addition, a separate sample of persons aged 18 to 29 years (N = 1894) was drawn using the same sampling design, of whom 1710 (90%) participated. A detailed comprehensive description of the methods and processes has been published elsewhere [17]–[19]. In Health 2000 study, the eligible sample consisted of 5106 subjects (3858 adults aged 30–64 years and 1248 young adults aged 18–29 years). The information on occupational titles and/or occupational exposures was available for 4918 (96%) subjects.

The Finnish National Work and Health Surveys (FWH Surveys) have been conducted every third year since 1997 and collected information on perceived working conditions and the health of the working-age population, For the 1997–2003 Surveys, random samples of subjects aged 25–64 years independent of their working status (e.g working, unemployed, retired or student) have been drawn from the Finnish population register. For the 2009 Survey a random sample of subjects aged 20–64 years was drawn from Finnish employment statistics. The sample size has varied between 2031 and 2355 persons from year to year with a response rate of 58–72% [20]. At each survey a phone number was not found for about 10–16% of subjects. The proportion of non-participants in each survey was slightly higher among men than women and among subjects aged 24–34 years than the older subjects. Age, gender, education, socio-economical status and occupational sector of the respondents were compared with the Census data. No major differences were found. Thus, the respondents to the FWH Surveys represent rather well the targeted population.

Since no systematic time trend in physical exposures was detected, the data from all five surveys were combined. Hence, the total number of the interviewed persons with information on occupation during 1997–2009 was 11326.

The characteristics of the study population are presented in Table S1.

### Occupational classification

Both in the H2000 Study and in the FWH Surveys, the occupations were classified on the 4-digit level (including few occupations coded with 5 digits) according to the Classification of Occupations 2001 by Statistics Finland, which is based on the International Standard Classification of Occupations (ISCO-88). The classification is based on ten categories of professional skills. In total, the classification includes 432 job titles coded with 4 or 5 digits. In the Health 2000 Study, the accurate job titles were not available for 32 subjects and these subjects were excluded from further analyses.

### Exposure information

In the H2000 Study, exposure to physical work load was assessed through face-to-face interviews with a validated questionnaire [21]. The respondents were asked if they were exposed (yes/no) to physical work load in their current job. The following exposures were assessed: heavy physical work, kneeling or squatting, manual lifting, carrying or pushing, driving a motor vehicle, working with hands above shoulder level, and working in forward bent position (Table S2).

In the FWH Surveys, exposure information was collected using computer-assisted telephone interviews (CATI). The following exposures were assessed using Likert-scale: physical heaviness of work, kneeling or squatting, lifting heavy loads with or without lifting devices, working with hands above shoulder level, and working in forward bent position (Table S2). When studying the validity of the developed JEM, the exposures were dichotomized. Physical heaviness of work was categorized as: 1–3 “light to moderate” and 4–5 “heavy” physical work load. Kneeling or squatting, working with hands above shoulder level, and working in forward bent position were categorized as: 1 “exposed”, 2–5 “unexposed”. Lifting heavy loads with or without lifting devices was categorized as: 0–2 “no heavy lifting” and 3–4 “heavy lifting”, respectively. The questions were modified from previously validated measures [22].

### Low back pain

In the FWH Surveys, data on low back pain were collected with an interview using the question: “Have you during the past month (30 days) had long-lasting or recurrent pain in the lumbar spine? (yes/no)”.

### Development of the job exposure matrix (JEM)

We developed a gender-specific matrix with exposure estimates at each intersection between rows (occupational groups) and columns (physical load exposures). The selection of exposures included in the matrix was based on the current knowledge of risk factors for low back disorders [8], [22], [23]. We also included exposures that increase the biomechanical load on the low back (such as arm elevation) or those that in combination with other known risk factors could be related to LBP (such as kneeling or squatting). Exposure information was based on interviews in the Health 2000 survey. The exposure estimates were calculated as the prevalence of exposures (as percentages) in each occupation which included at least 20 subjects in order to obtain reliable estimates. The job titles with a small number (<20) of respondents were grouped based on the similarities of these job titles with regard to work tasks and exposures. The grouping of occupations was made by experts. A detailed description of the matrix development is presented in Appendix S1. We also used two alternative strategies to define occupational groups: (1) based on 3-digit occupational codes (3-digit JEM) and (2) based on 1-digit occupational codes (1-digit JEM).

Exposure estimates of the matrix were dichotomized: If at least 50% of workers in an occupation or occupational group were exposed, then the exposure estimate was set at 1 but otherwise at 0. As an alternative dichotomization, we used 40% cut-off-point to define exposed and non-exposed.

The Health 2000 Study and the Finnish National Work and Health Surveys have all obtained ethical approval from the appropriate ethics committees.

### Data analyses

To evaluate which of the occupational grouping strategies and, similarly, which cut-off points of dichotomization will optimize the JEM, the matrix performance was examined using four indicators of agreement (accuracy, kappa, sensitivity, and specificity). Accuracy was defined as degree of closeness of measurement to its actual value and kappa value as the chance-corrected measure of agreement between two methods. The kappa (κ) values were classified according to Cohen [24] (<0.2 poor, 0.20–0.40 fair, 0.40–0.60 moderate, 0.60–0.80 good, and 0.80–1.00 excellent). Sensitivity (ability of the test to identify positive results) and specificity (ability of the test to identify negative results) were calculated to measure agreement between the binary group-based (JEM) measures and the individual-based exposures.

Accuracy (ACC), sensitivity (SNS) and specificity (SPC) were calculated as following:
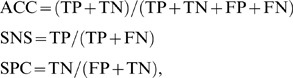
where TP – true positive, TN-true negative, FP- false positive, FN-false negative.

The higher the values are for accuracy, kappa, sensitivity and specificity, the better is the matrix performance. Furthermore, the JEM is optimized when the specificity is favored over sensitivity [25], [26].

The matrix validity was tested comparing the JEM measures with individual-based exposure measures from the FWH Surveys using the following indicators of agreement: accuracy, kappa, sensitivity, and specificity.

In addition, the associations between JEM estimates and LBP (predictive validity) were evaluated. Logistic regression analyses with age adjusted odds ratios (OR) and 95% confidence intervals (CIs) were carried out to study the associations between the JEM measures and low-back pain, as well as between individual-based measures and low-back pain.

All analyses were performed separately for men and women. Statistical analyses were performed using SAS version 9.1.

## Results

### Individual-based exposures

In the H2000 Study, men more frequently than women reported high physical exposures (Table S1). The largest gender difference was found in exposure to whole body vibration. In the FWH Surveys, the prevalence of exposure to heavy physical work, heavy lifting, arm elevation, and awkward trunk posture was lower than in H2000 study. The gender difference in exposures was less evident.

### Job exposure matrix

In the H2000 study, the final sample included 4886 subjects. Out of 432 possible job titles altogether 371 (86%) were recorded (Table 1). There were 68 job titles with at least 20 subjects. These job titles covered 62.7% of the study sample.

**Table 1:** Distribution of job titles in the Health 2000 Study.

Number of subjects per job titles	Number of job titles	% of job titles	Number of subjects	% of subjects
<10	238	64.2	911	18.6
11–19	65	17.5	913	18.7
>20	68	18.3	3062	62.7
Total	371	100	4886	100

Of the 303 job titles with a small number of respondents (<20), 280 were grouped. Still, 23 job titles (e.g. midwives, travel attendants and travel stewards, fishery workers, hunters and trappers) could not be included in any of them.

**Figure 1:**
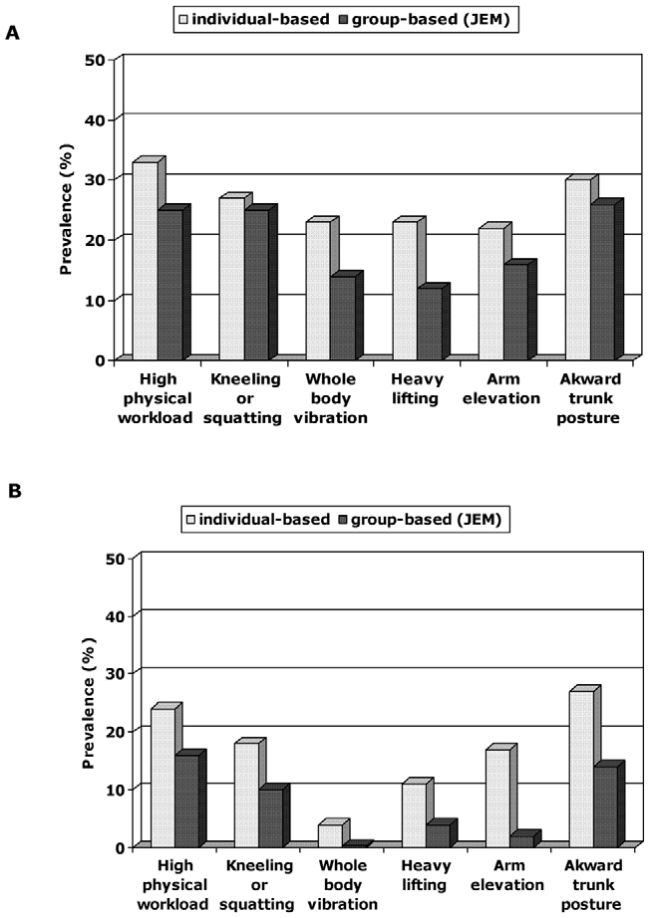
Prevalence and 95% CI of individual-based exposures and group-based exposures estimated by JEM in the Health 2000 Study. A: Among men; B: among women.

In both genders, the prevalence of group-based binary (using prevalence of 50% as the cut-off point) exposures was - as expected - lower than the prevalence of individual-based exposures (Figure 1). For some exposures, e.g. heavy lifting in both genders and arm elevation in women, the JEM showed a considerably lower proportion of occupations being exposed, suggesting a fairly large between-worker variance of these exposures within the occupation.

In men, the JEM with occupational groups based on the similarities of the job titles, tasks, or other features of the work environment (constructed JEM) and the JEM with occupational groups based on the 3-digit occupational codes (3-digit JEM) performed similarly in regard to accuracy, kappa, sensitivity and specificity for the majority of the exposures (Table 2). However, the constructed JEM performed slightly better than the 3-digit JEM for heavy lifting and arm elevation. The JEM with occupational groups based on the 1-digit occupational codes (1-digit JEM) tended to have higher sensitivity but lower specificity as compared to the constructed JEM. In women, a substantial drop in the performance of the 3-digit JEM was observed for kneeling or squatting, heavy lifting and awkward trunk posture. The 1-digit JEM had no estimates for three exposures (whole body vibration, heavy lifting and arm elevation), because for these exposures the prevalence was far below the cut-off point.

**Table 2:** Agreement of th e constructed job exposure matrix (JEM), 3-digit JEM and 1-digit JEM with individual-based measures in Health 2000 Study.

		Men				Women			
		Accuracy	Kappa	Sensit.	Specif.	Accuracy	Kappa	Sensit.	Specif.
Heavy physical work	Constructed JEM	0.80	0.52	0.57	0.91	0.81	0.41	0.44	0.93
	3-digit JEM	0.79	0.49	0.55	0.91	0.80	0.40	0.48	0.90
	1-digit JEM	0.75	0.45	0.66	0.80	0.79	0.31	0.31	0.94
Kneeling/squatting	Constructed JEM	0.83	0.53	0.56	0.93	0.85	0.35	0.35	0.96
	3-digit JEM	0.83	0.53	0.58	0.92	0.82	0.17	0.13	0.98
	1-digit JEM	0.81	0.53	0.70	0.85	0.83	0.17	0.13	0.98
Whole body vibration	Constructed JEM	0.88	0.60	0.54	0.98	0.97	0.17	0.09	1.00
	3-digit JEM	0.86	0.59	0.60	0.95	0.97	0.14	0.08	1.00
	1-digit JEM	0.80	0.42	0.49	0.90	-	-	-	-
Heavy lifting	Constructed JEM	0.79	0.28	0.30	0.94	0.90	0.26	0.20	0.98
	3-digit JEM	0.79	0.26	0.25	0.95	0.89	0.07	0.05	0.99
	1-digit JEM	0.77	0.16	0.16	0.96	-	-	-	-
Arm elevation	Constructed JEM	0.84	0.47	0.51	0.93	0.84	0.16	0.08	0.99
	3-digit JEM	0.83	0.41	0.40	0.95	0.84	0.13	0.09	0.99
	1-digit JEM	0.81	0.44	0.58	0.87	-	-	-	-
Awkward trunk posture	Constructed JEM	0.79	0.48	0.58	0.88	0.76	0.28	0.31	0.92
	3-digit JEM	0.78	0.46	0.57	0.87	0.73	0.18	0.21	0.93
	1-digit JEM	0.77	0.46	0.62	0.84	0.73	0.06	0.07	0.98

When 40% was used as the cut-off-point, the kappa values and sensitivity increased, particularly for heavy lifting in men (κ = 0.39, sensitivity = 0.56), and for the kneeling or squatting (κ = 0.46, sensitivity = 0.63) and arm elevation (κ = 0.26, sensitivity = 0.25) in women.

### Validity of the JEM

Agreement between the group-based (JEM) exposures and the individual-based exposures from the FWH Surveys is presented in Table 3. The agreement assessed by kappa was, in general, better among men than among women. In men, it was moderate for heavy physical work, poor for heavy lifting, and fair for all other exposures. In women, the agreement was moderate for heavy lifting, poor for arm elevation and fair for the other exposures. Specificity ranged from 0.84 to 0.92 in men, and from 0.91 to 0.98 in women. Sensitivity was lowest for heavy lifting in men and for arm elevation in women.

**Table 3:** Agreement between job exposure matrix (group-based) binary measures and individual-based measures in the Finnish National Work and Health Surveys.

Exposure	Men				Women			
	Accuracy	Kappa	Sensitivity	Specificity	Accuracy	Kappa	Sensitivity	Specificity
Heavy physical work	0.79	0.40	0.51	0.88	0.80	0.33	0.35	0.93
Kneeling/squatting	0.84	0.36	0.54	0.88	0.87	0.27	0.31	0.94
Heavy lifting	0.83	0.12	0.18	0.92	0.93	0.47	0.42	0.98
Arm elevation	0.85	0.38	0.55	0.89	0.89	0.18	0.13	0.97
Awkward trunk posture	0.78	0.36	0.53	0.84	0.76	0.23	0.30	0.91

### Predictive validity of the JEM

In the FWH Surveys, one-month prevalence of low back pain was slightly higher among women (29%) than men (26%). Associations between all individual-based exposures and low back pain were statistically significant in both sexes (Table 4). All odds ratios for group-based (JEM) exposures were lower than odds ratios for individual-based exposures with the exception of heavy lifting among women. Substantial reduction in odds ratios - also with loss of statistical significance – was observed for exposures to awkward trunk posture and arm elevation among women.

**Table 4:** Associations of the individual-based measures of exposures and the group-based (JEM) binary measures with one-month prevalence of low back pain in the Finnish National Work and Health Surveys.

	Men		Women	
Exposure	Individual-based	JEM	Individual-based	JEM
	OR (95% CI)	OR (95% CI)	OR (95% CI)	OR (95% CI)
Heavy physical work	2.11 (1.86–2.39)	1.61 (1.40–1.86)	2.04 (1.80–2.31)	1.67 (1.35–2.07)
Heavy lifting	1.67 (1.39–2.01)	1.57 (1.31–1.89)	1.47 (1.19–1.82)	2.08 (1.22–3.54)
Awkward trunk posture	2.22 (1.95–2.53)	1.57 (1.37–1.80)	1.85 (1.63–2.10)	1.08 (0.87–1.35)
Arm elevation	1.74 (1.48–2.05)	1.37 (1.17–1.60)	1.57 (1.33–1.85)	1.05 (0.71–1.56)
Kneeling or squatting	1.56 (1.30–1.87)	1.36 (1.17–1.58)	1.53 (1.26–1.85)	1.41 (1.15–1.74)

Age adjusted odds ratios (OR) and their 95% confidence intervals (95% CI).

## Discussion

We have constructed and validated a gender-specific job exposure matrix for physical exposures. The matrix was specifically designed for use in epidemiological studies of low back disorders. The exposure axis of the matrix included seven physical exposures. The occupation axis of the matrix was based on the original job titles or occupational groups. The matrix showed high specificity, especially among women. The validity of the matrix measured by kappa-statistic was fair for most exposures. The validity of the matrix was lower for heavy lifting among men and for arm elevation among women.

The developed matrix is based on national data, which represent well the Finnish adult population, including the distribution of occupations. The sample size was large enough to enable us to develop a gender-specific job exposure matrix and to keep several job titles unmerged. The developed matrix includes exposure estimates for 348 job titles that represent 81% of all possible job titles in Finland around the year 2000. Only a few rare job titles were not included in the matrix.

The exposure estimates of the matrix variables are based on self-reported information. Such an approach has been utilized in developed matrices for psychosocial and physical factors also earlier [27]–[29]. Generic job exposure matrices for chemical and microbiological exposures have usually been based on experts' judgments [30]. The expert judgment approach has been criticized for lower validity as compared to direct measurements of exposure [30], [31]. Obviously, the validity of the expert judgment approach depends on the type of exposure and the knowledge of the experts. On the other hand, direct measurements may be criticized due to small and unrepresentative samples.

The exposure information in this study was collected through face-to-face interviews using well-tested questions [21]. Self-reports on work-related physical factors are fairly reliable, especially when occupational activities have been classified dichotomously (exposed: yes/no) [32].

In our study, information on each physical exposure was collected with such dichotomous response options. Moreover, in order to obtain some quantitative information on the exposures, the present knowledge of the threshold values for potentially harmful levels of exposure (e.g., duration, weights lifted) was embedded in the dichotomous questions.

The occupational data used in this study were based on self-reported job titles. It has been proposed that self-reported job titles may be too common (e.g. manager, researcher) and, therefore, may not provide specific enough information for coding [33]. Because the current data were collected through interviews and the job titles were coded at the same time by experienced interviewers, more detailed information could be requested at once, if needed. Another source of limitation may, however, be the classification used: if the classification has not been recently updated, it may be somewhat incomplete due to the rapid changes in occupational titles. In this study, the occupational classification and collected data originated from the same time period. In addition, to reduce possible misclassification errors, the reported job titles were compared with information on educational level.

Twenty subjects with the same job title have been suggested to be used as a minimum number in a reliable estimation of exposure prevalence. In this study, merging of the job titles with a small number of respondents was performed very carefully by taking into account the work tasks and within-job variability of the exposures. We also evaluated whether the matrix estimates would change substantially if the job titles with at least 10 subjects would be kept separately. No significant differences in the JEM estimates were found (data not shown). This is in accordance with a previous study showing that consistent results could be attained with at least 10 respondents [34].

In epidemiological studies with no individual exposure information, job title is used as a proxy for exposure. Besides calculating risk for each possible occupation, researchers often group occupations into classes that correspond to 1-digit occupational classification. Such grouping strategy usually leads to biased risk estimates. In an earlier developed Finnish job-exposure matrix (FINJEM), which among other exposures includes, also physical exposures, the occupational axis consisted of unique job titles aggregated to 3-digit level [15]. We applied another strategy to define the occupational groups. In our matrix, groups were created based on the similarities of the job titles, tasks, or other features of the work environment the workers shared. The use of such modified grouping strategy increased matrix specificity as compared with grouping based on aggregation of occupations at 3-digit or 1-digit levels.

The use of 50% cut-off-point to define the exposed and the non-exposed is a common practice for constructing JEM (group-based) binary measures. Lowering of the cut-off-point to 40% resulted in noticeable gain in sensitivity without loss in specificity for rare exposures (e.g. whole body vibration, arm elevation), while for common exposures (e.g. heavy physical work, awkward trunk posture), the gain in sensitivity was accompanied by substantial loss in specificity. Hence, it could be suggested that, in case of less prevalent exposures, a lower cut-off-point could be used.

We evaluated the validity of the matrix in a representative Finnish population sample by comparing the group-based (JEM) measures with the individual-based measures; this has been a common approach to test validity also in previous studies [28], [30], [35]–[37]. Although the accuracy values between the measures were high in our study, the chance-corrected kappa values were lower. Kappa-statistic is sensitive to the prevalence of the studied phenomenon; therefore it should be interpreted with caution [38]. Too low or too high prevalence will result in a substantial reduction in kappa values, which can be misleading.

The sensitivity and specificity of the JEMs are usually evaluated against self-reports, even if it is well known that the self-reported exposures may be subject to recall bias. Study subjects with LBP may be especially prone to overestimate their exposure to physical work load [32]. Moreover, the magnitude of the possible overestimation could vary depending on the type of exposure. In this study, relatively low validity of JEM for some exposures could reflect this differential misclassification of exposures. In addition, between-worker and within-worker variances within an occupation could be another reason for low sensitivity and specificity. It should be kept in mind that the JEM is a rather crude exposure measure and, is therefore useful for establishing the importance of particular risk factors in large population surveys. However it is not as well suited for establishing exposure-response relationships that are informative for deriving cut-off values for safe work. In estimating exposure at the group-level, specificity has been considered more important than sensitivity [25], [26]. High specificity of JEM measures observed in the current study suggests that the constructed JEM can be applied to other populations as well.

In the FWH Surveys, almost every third respondent reported low back pain during the previous month - women somewhat more frequently than men - which is in line with previously published research [39]. We compared whether the associations between the group-based (JEM) measures and low back pain differed from those seen between individual-based measures and low back pain. The associations observed between the JEM estimates and LBP were in line with the previously published findings [7], [8]. However, some of the associations were attenuated and lost their significance as compared with those found for individual-based exposures. JEMs have often been criticized for potential non-differential misclassification of exposures that results in attenuation of the association between exposure and outcome. However, Tielemans et al. [40] showed that an individual-based exposure assessment generates precise though biased estimates, while a group-based assessment generates less precise but unbiased estimates.

## Conclusions

We constructed a gender-specific JEM for physical exposures to be used in large-scale epidemiological studies, in which more precise but more labor-intensive methods are often not feasible. The developed matrix showed relatively high specificity without compromising sensitivity. The matrix was based on Finnish data and is therefore intended primarily for national use. We, however, foresee that it could be applicable, with some modifications, in other countries with a similar level of technology. In occupational health practice, the matrix may be useful in characterization of exposures of worker groups and in identification of high-risk occupational groups.

## Supporting Information

Table S1 **Information on data collection and characteristics of the study populations.**
(DOC)

Table S2 **Exposure assessment in the Health 2000 Study and in the Finnish National Work and Health Surveys.**
(DOC)

Appendix S1 **Development of the matrix.**
(DOC)
