# Supplementary material for: Development and Validation of a Job Exposure Matrix for Physical Risk Factors in Low Back Pain
Source: PLoS One. 2012 Nov 12;7(11):e48680. doi: 10.1371/journal.pone.0048680 (PMC3495969; doi:10.1371/journal.pone.0048680)
Supplement: Table S1 — Information on data collection and characteristics of the study populations. (DOC) [file pone.0048680.s001.doc]

Table S1. Information on data collection and characteristics of the study populations

|  | | Health 2000 Study | | | The Finnish National Work and Health Survey | | |
| --- | --- | --- | --- | --- | --- | --- | --- |
| Data collected in | | 2000-2001 | | | 2000, 2003, 2006, 2009 | | |
| Data collected with | | Interview, questionnaires | | | Computer-assisted telephone interview (CATI) | | |
| Job classification | | 2001 (based on ISCO-88) | | | 2001 (based on ISCO-88) | | |
|  | | Men | Women | Total | Men | Women | Total |
| Number of subjects (%) | | 2 437 (49.6) | 2 481  (50.4) | 4 918 | 5 684 (50.1) | 5 642 (49.8) | 11 326 |
| Age (mean ± SD; min, max) | | 41±11 (18,64) | 41±11 (18,64) | 41±11 (18,64) | 43±10 (20,64) | 44±10 (20,64) | 44±10 (20,64) |
| Age group (%) | |  |  |  |  |  |  |
|  | 18-29 years | 18.7 | 16.7 | 17.7 | 11.7 | 9.0 | 10.3 |
|  | 30-39 years | 27.9 | 27.9 | 27.9 | 25.7 | 23.6 | 24.7 |
|  | 40-49 years | 28.8 | 30.2 | 29.5 | 32.1 | 33.3 | 32.7 |
|  | 50-64 years | 24.5 | 25.2 | 24.8 | 30.5 | 34.1 | 32.3 |
| Education^a^ (%) | |  |  |  |  |  |  |
|  | Basic | 28.7 | 24.7 | 26.7 | 20.8 | 20.1 | 20.5 |
|  | Intermediate | 41.7 | 29.4 | 35.5 | 41.6 | 29.1 | 35.4 |
|  | Collage | 15.4 | 28.3 | 21.9 | 18.9 | 29.5 | 24.2 |
|  | High | 14.2 | 17.6 | 15.8 | 18.6 | 21.3 | 20.0 |
| Employer sector^b^ (%) | |  |  |  |  |  |  |
|  | Private | 69.7 | 47.3 | 57.9 | 77.0 | 52.5 | 65.6 |
|  | Local government | 12.5 | 38.5 | 26.2 | 10.4 | 37.6 | 21.1 |
|  | Central government | 15.2 | 10.4 | 12.7 | 8.8 | 7.1 | 9.8 |
|  | Other | 2.5 | 3.8 | 3.2 | 3.8 | 2.8 | 3.3 |
| Occupational group (%) | |  |  |  |  |  |  |
|  | Employed | 83.2 | 90.4 | 86.8 | 82.3 | 90.9 | 86.6 |
|  | Self-employed^c^ | 16.8 | 9.6 | 13.2 | 16.7 | 9.1 | 13.4 |
|  |  |  |  |  |  |  |  |
| Socioeconomic group^d^ (%) | |  |  |  |  |  |  |
|  | Farmer | 4.0 | 2.3 | 3.1 | 4.7 | 2.2 | 3.5 |
|  | Self-employed | 12.5 | 7.1 | 9.8 | 12.9 | 6.9 | 9.9 |
|  | Manual workers | 41.6 | 15.4 | 28.3 | 39.4 | 18.7 | 29.1 |
|  | Lower-level employees | 24.1 | 26.7 | 25.4 | 24.6 | 22.6 | 23.6 |
|  | Upper-level employees | 17.8 | 48.6 | 33.4 | 18.3 | 49.6 | 33.9 |
| Occupational sector | |  |  |  |  |  |  |
|  | Agriculture, forestry, fishing and hunting | 6.8 | 3.3 | 5.0 | 7.0 | 3.3 | 5.2 |
|  | Manufacturing | 29.1 | 10.4 | 19.5 | 27.6 | 12.2 | 19.9 |
|  | Construction | 11.8 | 1.0 | 6.4 | 10.8 | 1.0 | 5.9 |
|  | Trade, accommodation and food services | 8.0 | 15.8 | 11.9 | 10.4 | 13.7 | 12.0 |
|  | Transportation and communications | 11.7 | 3.4 | 7.5 | 10.6 | 3.9 | 7.3 |
|  | Finance and insurance | 1.2 | 3.3 | 2.3 | 1.4 | 3.4 | 2.4 |
|  | Public administration | 5.9 | 6.3 | 6.1 | 4.3 | 5.9 | 5.1 |
|  | Education | 3.5 | 9.6 | 6.6 | 5.0 | 10.7 | 7.8 |
|  | Health care and social sector | 3.4 | 27.3 | 15.4 | 3.7 | 28.1 | 15.9 |
|  | Other services | 15.3 | 17.4 | 16.3 | 4.8 | 7.1 | 6.0 |
| individual-based exposures | |  |  |  |  |  |  |
|  | Heavy physical work | 32.9 | 23.9 | 28.5 | 25.3 | 25.3 | 25.3 |
|  | Kneeling or squatting | 26.7 | 18.0 | 22.5 | 13.0 | 11.1 | 12.0 |
|  | Whole body vibration | 23.3 | 3.7 | 13.8 | - | - | - |
|  | Heavy lifting | 23.1 | 10.8 | 17.1 | 13.1 | 9.2 | 11.1 |
|  | Arm elevation | 21.5 | 17.2 | 19.4 | 12.4 | 11.8 | 12.1 |
|  | Awkward trunk posture | 30.6 | 27.4 | 29.0 | 22.5 | 24.8 | 23.6 |

^a^ Basic: high school or less and vocational courses or no vocational education; intermediate: any basic education and vocational school; collage: apprenticeship contract or a special vocational qualification; high: higher vocational qualification or a university degree

^b^ Local government: municipalities; central government: state; other sectors: churches, non-profit sector

^c^ Self-employed: farmers, small employers, other employers etc.

^d^ Farmer: workers in agriculture; self-employed; manual workers: workers in forestry, commercial fishing, manufacturing, distribution, and service; lower-level employees: administrative and clerical occupations; upper-level employees: administrative, managerial, professional and related occupations

Table S1.2. Exposure assessment in the Health 2000 Study and in the Finnish National Work and Health Surveys

|  | Health 2000 | | The Finnish National Work and Health | |
| --- | --- | --- | --- | --- |
| Exposure | Question | Response categories | Question | Response categories |
| Heavy physical work | Is your current job physically demanding involving e.g. lifting and carrying heavy loads, excavating, shovelling, or hammering? | yes / no | Is your work physically | 1) light,  2) fairly light,  3) somewhat demanding,  4) fairly demanding,  5) very demanding |
| Kneeling or squatting | Does your current job involve kneeling or squatting for at least one hour a day? | yes / no | Does your work involve working on bended knee or squatted down | 1) daily at least 1-2 h,  2) every day for less than 1 h,  3) almost every day,  4) occasionally,  5) not at all |
| Driving a motor vehicle ("Whole body vibration") | Does your current job involve driving a car, tractor or other motor vehicle for at least four hours a day? | yes / no |  |  |
| Manual lifting, carrying or pushing ("Heavy lifting") | Does your current job involve manual lifting, carrying or pushing items heavier than 20 kg:s at least 10 times every day? | yes / no | Do you use lifting devices when lifting heavy loads (>25 kg) | 0) do not lift heavy loads at all,  1) always,  2) sometimes,  3) do not use at all, although available,  4) do not use at all, not available |
| Working with hands above the shoulder level ("Arm elevation") | Does your current job involve working with hands above shoulder level for at least one hour a day? | yes / no | Does your work involve holding upper limb (s) above shoulder level | 1) daily at least 1-2 h,  2) every day for less than 1 h,  3) almost every day,  4) occasionally,  5) not at all |
| Working in a forward bent posture ("Awkward trunk posture") | Does your current job involve working in a forward bend position (while standing or kneeling) for at least one hour a day? | yes / no | Does your work involve holding your back bent forward or in awkward posture | 1) daily at least 1-2 h,  2) every day for less than 1 h,  3) almost every day,  4) occasionally,  5) not at all |
