# Supplementary material for: Development and Validation of a Job Exposure Matrix for Physical Risk Factors in Low Back Pain
Source: PLoS One. 2012 Nov 12;7(11):e48680. doi: 10.1371/journal.pone.0048680 (PMC3495969; doi:10.1371/journal.pone.0048680)
Supplement: Table S2 — Exposure assessment in the Health 2000 Study and in the Finnish National Work and Health Surveys. (DOC) [file pone.0048680.s002.doc]

Table S2. Exposure assessment in the Health 2000 Study and in the Finnish National Work and Health Surveys

|  | Health 2000 | | The Finnish National Work and Health | |
| --- | --- | --- | --- | --- |
| Exposure | Question | Response categories | Question | Response categories |
| Heavy physical work | Is your current job physically demanding involving e.g. lifting and carrying heavy loads, excavating, shovelling, or hammering? | yes / no | Is your work physically | 1) light,  2) fairly light,  3) somewhat demanding,  4) fairly demanding,  5) very demanding |
| Kneeling or squatting | Does your current job involve kneeling or squatting for at least one hour a day? | yes / no | Does your work involve working on bended knee or squatted down | 1) daily at least 1-2 h,  2) every day for less than 1 h,  3) almost every day,  4) occasionally,  5) not at all |
| Driving a motor vehicle ("Whole body vibration") | Does your current job involve driving a car, tractor or other motor vehicle for at least four hours a day? | yes / no |  |  |
| Manual lifting, carrying or pushing ("Heavy lifting") | Does your current job involve manual lifting, carrying or pushing items heavier than 20 kg:s at least 10 times every day? | yes / no | Do you use lifting devices when lifting heavy loads (>25 kg) | 0) do not lift heavy loads at all,  1) always,  2) sometimes,  3) do not use at all, although available,  4) do not use at all, not available |
| Working with hands above the shoulder level ("Arm elevation") | Does your current job involve working with hands above shoulder level for at least one hour a day? | yes / no | Does your work involve holding upper limb (s) above shoulder level | 1) daily at least 1-2 h,  2) every day for less than 1 h,  3) almost every day,  4) occasionally,  5) not at all |
| Working in a forward bent posture ("Awkward trunk posture") | Does your current job involve working in a forward bend position (while standing or kneeling) for at least one hour a day? | yes / no | Does your work involve holding your back bent forward or in awkward posture | 1) daily at least 1-2 h,  2) every day for less than 1 h,  3) almost every day,  4) occasionally,  5) not at all |
