# Supplementary material for: Development and Validation of a Job Exposure Matrix for Physical Risk Factors in Low Back Pain
Source: PLoS One. 2012 Nov 12;7(11):e48680. doi: 10.1371/journal.pone.0048680 (PMC3495969; doi:10.1371/journal.pone.0048680)
Supplement: Appendix S1 — Development of the matrix. (DOC) [file pone.0048680.s003.doc]

**APPENDIX S1. Development of the matrix.**

Reviewing the data and grouping of the occupations

The occupations with smaller number (< 20) of respondents were grouped. In order to decide, which occupations could form a group, the exposure information was carefully reviewed by experts and groups were created based on the similarities of the work tasks (e.g. car, taxi, and van drivers and heavy truck and lorry drivers) and similarities of the exposures. Information on occupations and work tasks were collected from the literature (e.g. from scientific articles and ergonomic guidebooks) and from Internet sources (e.g. from Statistics Finland's descriptions of the occupations, O'NET*, and job advertisements). In addition, occupations were reviewed by Finnish Institute of Occupational Health experts.

We noticed that self-employed workers had even higher risk for physical exposures than manual workers, suggesting that many self-employees perform regularly manual tasks. Hence, e.g. managers of small enterprises in the construction and building industry were merged, because often the main tasks of the managers are rather similar to those of workers in the company.

After merging the occupations with few respondents there were still some occupational groups with less than 20 subjects. The exposure estimates of those groups were calculated using the following methods:

1. In female- or male-dominated occupations exposure estimates for the non-dominant gender were based on the total number of respondents assuming that both genders perform similar work tasks
2. The occupational groups with similar physical exposures were merged

Selected examples of the new classification of occupations are shown in Table 1.

To justify the chosen grouping strategy of the occupations, we also constructed JEMs based on the 3-digit and 1-digit occupational codes.

Examples of the occupational groupings

| Occupations that were not grouped | |
| --- | --- |
|  | Primary education teaching professionals |
|  | Shop, stall and market salespersons and demonstrators |
|  | Crop and animal producers and workers |
| Occupations with similar work tasks that were merged | |
|  | Building and fire inspectors  Safety, health and quality inspectors |
|  | Parish clergy and other religious professionals  Deacons, deaconesses and religious associate professionals |
|  | Transport conductors,  Travel guides and tour leaders |
|  | Telecommunications installers and mechanics  Electrical line installers, repairers and cable jointers |
|  | Motorised farm and forestry plant operators  Earth-moving and related plant operators |
| Occupations, where responses of the both genders were merged | |
|  | Housekeepers and related supervisors |
|  | Hairdressers, barbers, beauticians and related workers |
|  | Waiters, waitresses and bartenders |
|  | Child-care workers |
|  | Painters and related workers |
